# Establishment of a model of sentinel lymph node metastasis using immunodeficient swine

**DOI:** 10.1038/s41598-019-44171-w

**Published:** 2019-05-28

**Authors:** Toshiaki Kurihara, Sachiko Matsuda, Yuki Nakamura, Shunichi Suzuki, Daiichiro Fuchimoto, Akira Onishi, Kohei Saeki, Takayuki Nakagawa, Reina Fujiwara, Masatoshi Kamata, Junko Kuramoto, Kaori Kameyama, Masaki Sekino, Moriaki Kusakabe, Tetsu Hayashida, Hiromitsu Jinno, Yuko Kitagawa

**Affiliations:** 1 Department of Surgery, Keio University School of Medicine, 35 Shinanomachi, Shinjuku, Tokyo 160-8582 Japan; 2 Division of Animal Sciences, Institute of Agrobiological Sciences, National Agriculture and Food Research Organization (NARO), Tsukuba, Ibaraki 305-0901 Japan; 3 Laboratory of Animal Reproduction, Department of Animal Science and Resources, College of Bioresource Sciences, Nihon University, Fujisawa, Kanagawa 252-0880 Japan; 4 Graduate School of Agricultural and Life Sciences, The University of Tokyo, 1-1-1 Yayoi, Bunkyo, Tokyo 113-8657 Japan; 5 Veterinary Medical Center, Graduate School of Agricultural and Life Sciences, The University of Tokyo, 1-1-1, Yayoi, Bunkyo, Tokyo 113-8657 Japan; 6 Department of Pathology, Keio University School of Medicine, 35 Shinanomachi, Shinjuku, Tokyo 160-8582 Japan; 7 Department of Diagnostic Pathology, Keio University Hospital, 35 Shinanomachi, Shinjuku, Tokyo 160-8582 Japan; 8 Graduate School of Engineering, The University of Tokyo, 7-3-1 Hongo, Bunkyo, Tokyo 113-8656 Japan; 9 Graduate School of Agricultural and Life Sciences, Research Center for Food Safety, The University of Tokyo, 1-1-1 Yayoi, Bunkyo, Tokyo 113-8657 Japan; 10 Matrix Cell Research Institute, Inc., 1-35-3 Kamikashiwada, 300-1232 Ushiku, Ibaraki Japan; 11 Department of Surgery, Teikyo University School of Medicine, 2-11-1 Kaga Itabashi, Tokyo, 173-8605 Japan

**Keywords:** Metastasis, Metastasis

## Abstract

Lymph node metastasis occurs via the migration of cancer cells through the lymphatic system. Sentinel lymph node (SLN) biopsy is a common diagnostic strategy. SLNs have been studied using healthy rodents and large animals without metastasis. Here we used immunodeficient swine to establish a model of lymph node metastasis. We used RAG2-knockout immunodeficient swine. A431 human epithelial carcinoma cells expressing green fluorescent protein were injected subcutaneously into the posterior sides of the auricle, forelimb and hindlimb of knockout swine. Indigo carmine dye was injected subcutaneously 8 weeks after tumour cell transplantation. SLNs were extracted, observed using a stereoscopic fluorescence microscope and analysed histologically using haematoxylin and eosin staining, and immunohistochemistry. Lymphoid follicles were found in wild-type swine, and a few aggregated lymphocytes and immature lymphoid follicles were observed in knockout swine. Fluorescence in the lymph nodes indicated metastasis of tumour cells to the lymph nodes. Tumour cells replaced lymph node architectures, showed high-grade nuclear atypia and formed irregular tumour nests. Our model may be useful for the preclinical validation of diagnostic methods and minimally invasive treatment of metastatic cancer.

## Introduction

Hundreds of lymph nodes are distributed throughout the body, forming small sac-like structures located along lymphatic vessels. Lymph nodes function as a barrier to infection and activate the immune response mediated by T and B lymphocytes^1^.

Cancer metastasis frequently occurs via migration of cancer cells through the lymphatic system^1^. Therefore, reliable dissection of regional lymph nodes is required for cancer surgery. The presence or absence of clinical or pathological lymph node metastasis greatly influences the selection of treatment such as expansion surgery, anti-cancer drug treatment and can predict a patient’s prognosis.

Sentinel lymph node biopsy (SLNB) is a common diagnostic method. Particularly in breast cancer, the results of SLNB reflect axillary lymph node metastasis^2–4^. Further, SLNB is significantly less likely to cause lymphedema or motor dysfunction of the upper limbs compared with axillary lymph node dissection^5^.

SLNB is the standard treatment for malignant melanoma. The 10-year disease-free and overall survival rates are significantly superior in the SLNB group compared with those of the lymph node nonresected group^6,7^. Research on SLNB applied to gastrointestinal cancer is progressing, and the results show promise for reducing the requirement for surgery and for improving the quality of life of patients^8^. However, SLNB was performed on patients with early-stage gastric cancer with an identification and correct diagnosis rates of 97.5% and 99%, respectively^8^.

SLNs are identified using radioisotopes (RIs), blue dyes, or a combination of both, although there are problems such as technical difficulties and limitations to the clinical use of RIs^9,10^. To overcome these problems, methods to identify SLNs using other substances such as indocyanine green and superparamagnetic nanoparticles have been used in clinical trials and in routine practice instead of conventional RIs and blue dye methods^11–13^.

Despite numerous studies published regarding SLNB^3–14^, many aspects must be addressed, such as accurate measurements of lymph flow, evaluation of particle sizes suitable for lymphatic vessel diameters and clinical medical policy based on the presence or absence of metastasis to SLNs. Further, methods are not available for accurate diagnosis of the presence or absence of metastasis without extracting SLNs nor are methods for treating lymph node metastasis without excision. Methods to identify SLNs in carcinomas other than breast cancer are under development.

Superparamagnetic iron oxide (SPIO) particles have been introduced as a contrast agent for magnetic resonance imaging (MRI) of head and neck cancer^15^, and SPIO-enhanced MRI detects sentinel lymph nodes and metastases^16,17^. However, sentinel lymph node metastases <2 mm of breast cancers are undetectable^18^. Therefore, techniques using these agents have not been fully translated to the clinic.

Studies of SLNs have been performed using healthy rodents or large animals with non-metastatic tumours^19–22^. We developed a rodent model of lymph node metastasis that enabled us to show that magnetic particles are useful for identifying SLNs^23^ and that photodynamic therapy is effective for treating lymph node metastasis^24,25^. However, the small size of rodents is insufficient for measuring lymph flow, applying novel imaging methods, determining the optimum dose of a therapeutic agent and the optimum treatment protocol. Therefore, a large animal model of lymph node metastasis is required. Tumour-bearing swine models are available^26^, although we are unaware of published studies that report establishing lymph node metastasis model.

Immunodeficient swine were developed using genetic engineering techniques to inactivate the recombination activating 2 gene (*RAG2*)^27^. This swine strain is immunodeficient and possesses macroscopically immature lymph nodes and thymus. Microscopic observations revealed that this swine stain lacks the thymic medulla, lymphoid aggregation in the spleen and lymphoid follicles in lymph nodes. Despite these limitations, these swine provided an adequate system for our present studies of SLNs.

## Results

### Lymph flow

**Figure 1:**
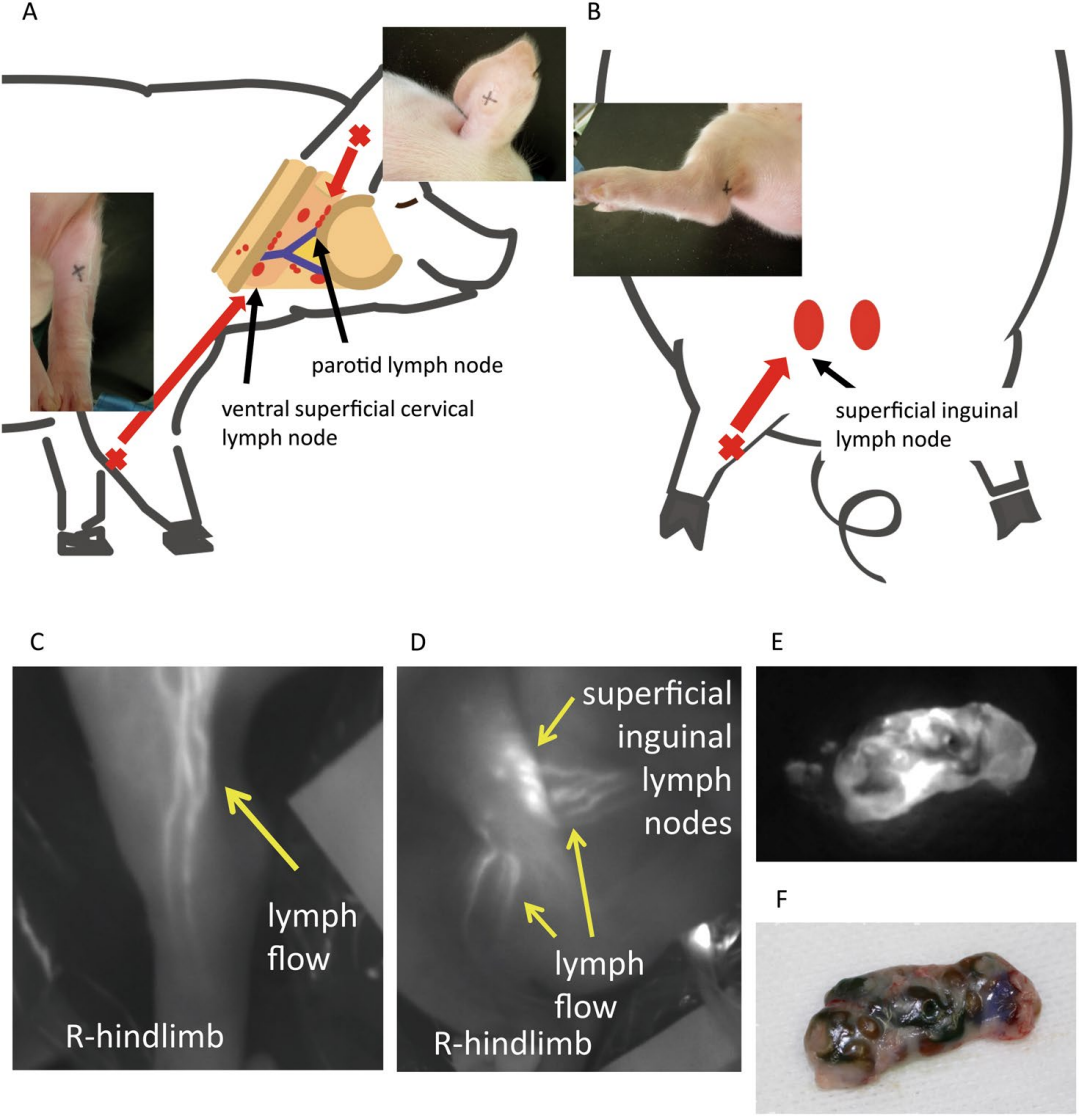
Lymph flow in swine. The lymph centre is formed by the inflowing lymph node group to which lymph flow is formed for each tributary area. (**A**) The parotid lymph centre receives lymph flow from the half-side region of the cranial dorsal side and orbital and masticatory muscles. The superficial cervical lymph centre receives lymph flow from the neck, chest and proximal regions of the forelimbs. (**B**) The iliofemoral lymph centre receives lymph flow from the flank, dorsal side of the abdominal wall, scrotum and mammary glands. (**C,D**) Lymph flow and sentinel lymph nodes detected using indocyanine green (ICG). The lymph flow from the right hindlimb to the superficial inguinal lymph nodes was visualized when illuminated with near-infrared light. Lymph nodes illuminated with near-infrared light (**E**) and white light (**F**).

The lymph centre is formed by the inflowing lymph node group to which lymph flow is directed from each tributary area^28^. A published procedure is available for clinical identification of sentinel lymph nodes strained with isosulfan blue^9,10^, and the modified method was used in our study. Here we subcutaneously injected tumour cells and indigo carmine dye or indocyanine green (ICG) into the posterior sides of the auricle, forelimb and hindlimb that flow into the parotid, superficial cervical, and iliofemoral lymph centres, respectively (Fig. 1A,B). The parotid lymph centre receives lymph flow from the half-side region of the cranial dorsal side and the orbital and masticatory muscles. The superficial cervical lymph centre receives lymph flow from the neck, chest and proximal regions of forelimbs. The iliofemoral lymph centre receives lymph flow from the flank, the dorsal side of abdominal wall, scrotum and mammary gland. The lymph node centre includes lymph nodes with lymph flow that always passes through from each peripheral site. After injection of ICG, the lymph flow from the right hindlimb to the superficial inguinal lymph nodes (Fig. 1C,D) and lymph nodes (Fig. 1E) was visualized when illuminated with near-infrared light. The same lymph nodes illuminated with visible light are shown Fig. 1F. Therefore, these centres served here as representative SLNs.

### Identification of SLNs in wild-type swine

**Figure 2:**
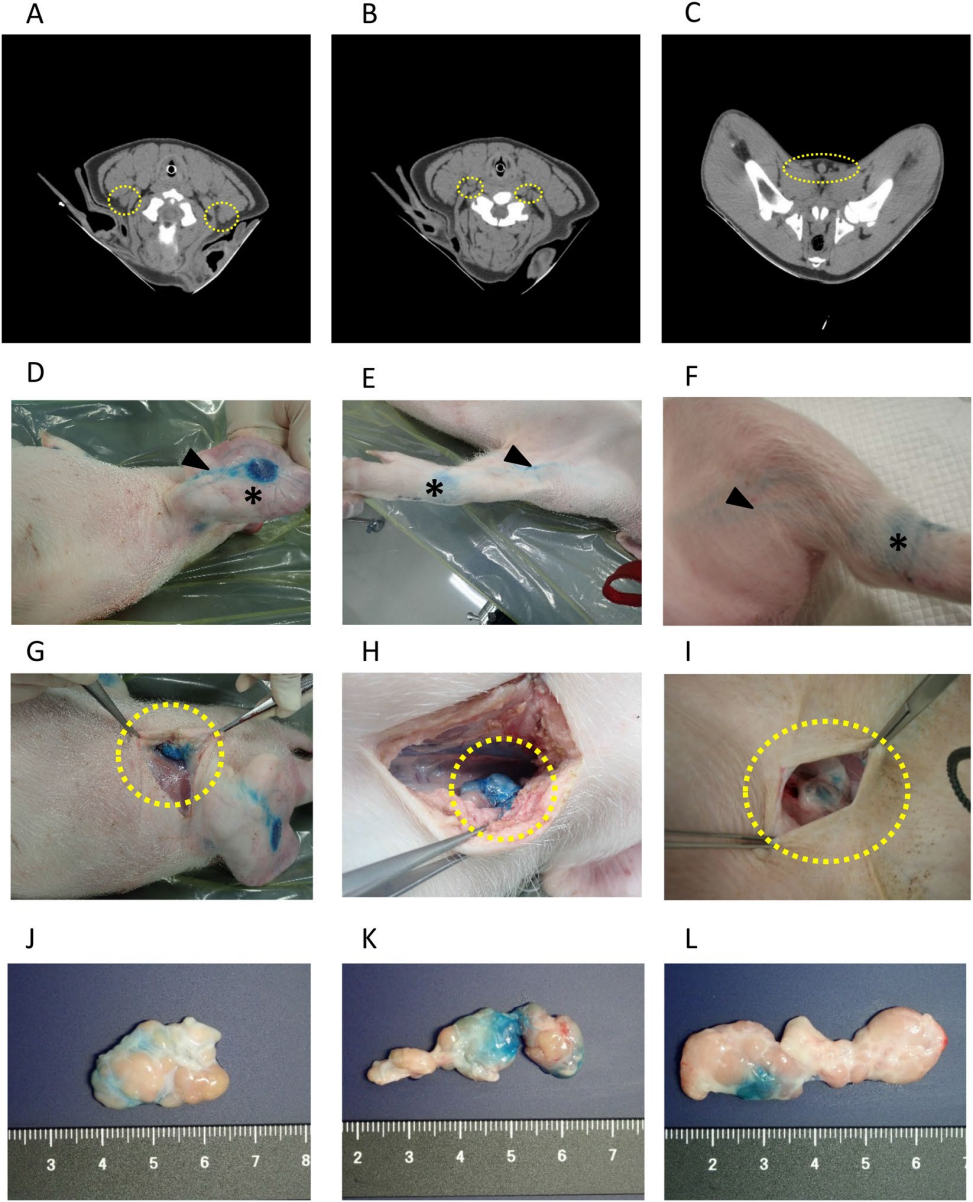
Lymph nodes of wild-type swine. (**A–C**) CT images of parotid lymph nodes (**A**), ventral superficial cervical lymph nodes (**B**), and superficial inguinal lymph nodes (**C**). (**D–F**) Lymph flow to parotid lymph nodes (**D**), ventral superficial cervical lymph nodes (**E**), and superficial inguinal lymph nodes (**F**). Asterisks indicate the injection sites of indigo carmine, and arrowheads indicate lymph flow. (**G–I**) Stained lymph nodes: parotid lymph nodes (**G**), ventral superficial cervical lymph nodes (**H**) and superficial inguinal lymph nodes (**I**). (**J–L**) Extracted lymph nodes: parotid lymph nodes (**J**), ventral superficial cervical lymph nodes (**K**) and superficial inguinal lymph nodes (**L**).

We used computed tomography (CT) to visualise SLNs of one swine. CT detected parotid lymph nodes, superficial cervical lymph nodes and superficial inguinal lymph nodes. However, the boundaries between parotid lymph nodes, superficial cervical lymph nodes and surrounding tissues such as the salivary gland were unclear. Therefore, it was difficult to identify these lymph nodes (Fig. 2A,B).

Superficial inguinal lymph nodes were close to the surface of the body (Fig. 2C). CT revealed flat inguinal lymph nodes that continued in a beaded shape with sizes of 17.9 × 8.0 mm and 17.2 × 8.0 mm on the right and left, respectively.

Lymphatic flow was detected by subcutaneously injecting indigo carmine (Fig. 2D–F). While general anaesthesia was maintained, an incision was made, and stained lymph nodes stained were observed (Fig. 2G–I). Each lymph node was stained with the dye (Fig. 2J–L).

### Identification of SLNs in knockout swine

**Figure 3:**
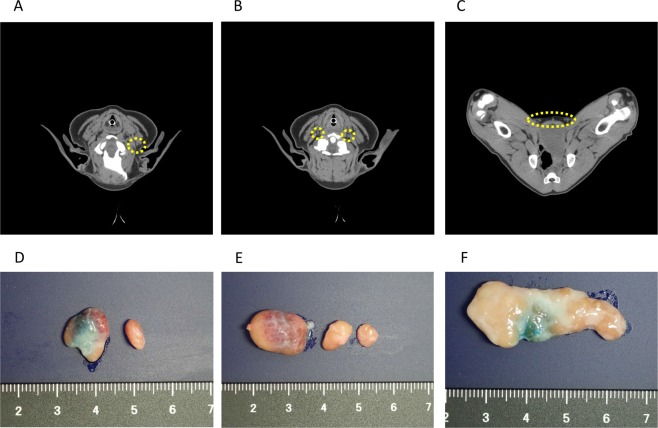
Lymph nodes in RAG2-knockout swine without tumours. (**A–C**) CT images of parotid lymph nodes (**A**), ventral superficial cervical lymph nodes (**B**) and superficial inguinal lymph nodes (**C**). (**D–F**) Extracted lymph nodes: parotid lymph nodes (**D**), ventral superficial cervical lymph nodes (**E**) and superficial inguinal lymph nodes (**F**).

CT imaging of a knockout swine without tumours revealed that its lymph nodes were smaller than those of the wild-type. Identification of the parotid lymph node and ventral superficial cervical lymph node was more difficult (Fig. 3A,B). The superficial inguinal lymph node was 14.6 mm × 6.4 mm on the right and 12.6 mm × 6.2 mm on the left (Fig. 3C). This lymph node, which was extracted using the dye method was smaller than that of the wild-type (Fig. 3D–F).

### Establishment of a model of metastasis and identification of SLNs

**Figure 4:**
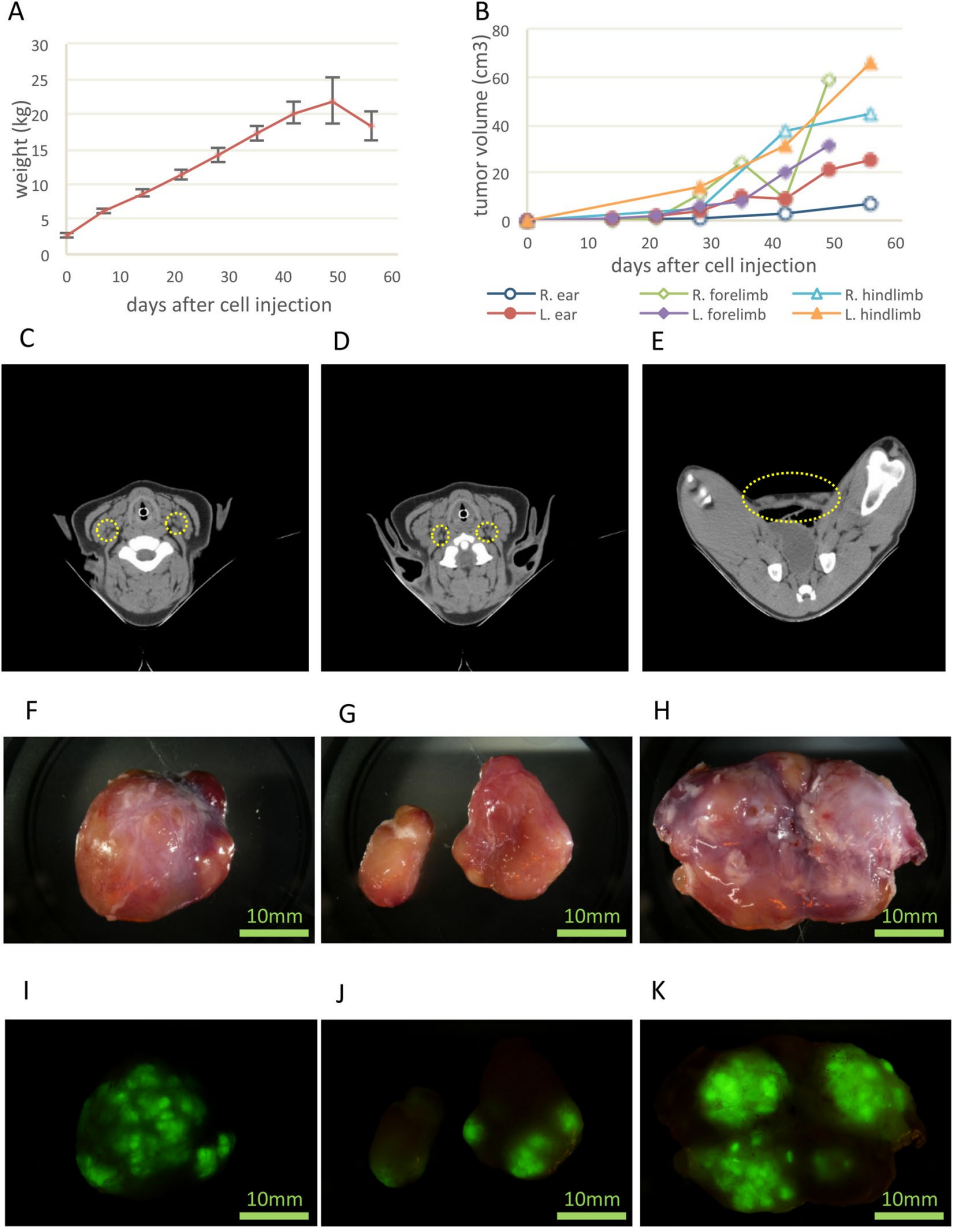
Transition of average body weight and the size of the primary tumour and lymph nodes in the metastatic model. (**A**) Transition of average body weight. The average weight decreased by week 7, because one swine was poorly conditioned and lost weight. (**B**) Transition of the size of the primary tumour at the auricle and limbs. (**C–E**) CT images of parotid lymph nodes (**C**), ventral superficial cervical lymph nodes (**D**) and superficial inguinal lymph nodes (**E**). (**F–H**) Extracted lymph nodes: parotid lymph nodes (**F**), ventral superficial cervical lymph nodes (**G**) and superficial inguinal lymph nodes (**H**). (**I–K**) Lymph nodes observed using a stereoscopic fluorescence microscope: parotid lymph nodes (**I**), ventral superficial cervical lymph nodes (**J**) and superficial inguinal lymph nodes (**K**). The scale bar = 10 mm (**F–K**).

The body weight of swine (Fig. 4A) and size of the primary tumour (Fig. 4B) increased after subcutaneous injection of A431-GFP cells. The tumour size of right forelimb was reduced at sixth week with unknown reason. Eight weeks after transplantation of A431 cells, CT did not distinguish the boundary of the parotid lymph nodes, and the ventral superficial cervical lymph nodes were difficult to discern (Fig. 4C,D). The shape of the superficial inguinal lymph nodes was more spherical and swollen than in the RAG2 knockout or wild-type. Their dimensions were 17.9 ± 6.0 mm × 11.0 ± 2.5 mm on the right and 16.7 ± 2.8 mm × 11.0 ± 0.2 mm on the left (Fig. 4E).

These lymph nodes were extracted using the same method employed for the control (Fig. 4F–H). The extracted lymph nodes emitted fluorescence, indicating metastasis of tumour cells to the lymph nodes (Fig. 4I–K). A431 cells were transplanted into six swine at 32 sites. Tumour growth was observed at 20 sites, and fluorescence was confirmed at 11 sites.

### Histological analysis of lymph nodes

**Figure 5:**
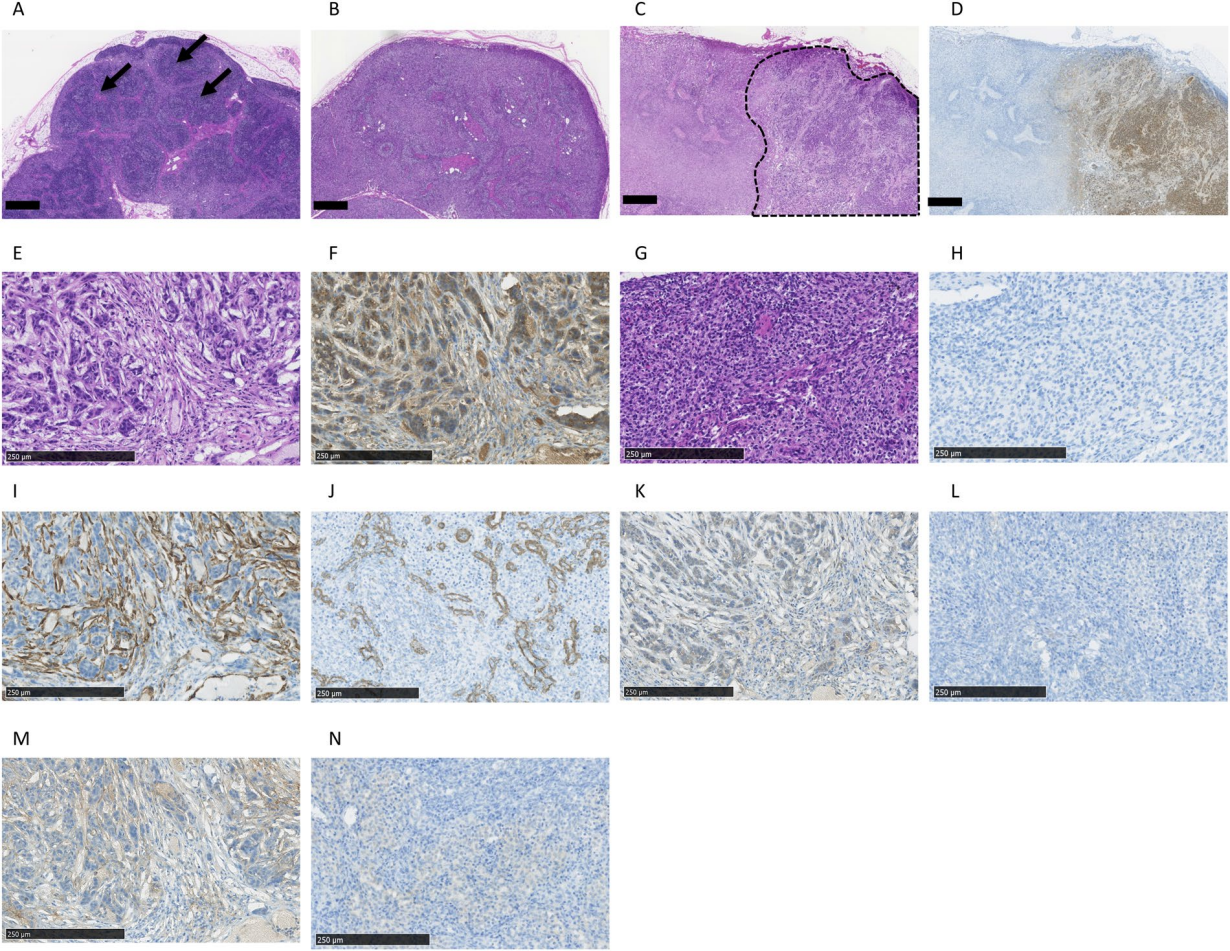
Histological analysis of lymph nodes. HE-stained lymph nodes of wild-type (**A**) and lymphoid follicles is indicated by the arrows. HE-stained lymph nodes of RAG2-knockout swine (**B**). HE-stained metastatic lymph nodes of RAG2-knockout swine (**C**). Metastatic tumour cells are surrounded by a dotted line. Immunohistochemical analysis of GFP expression in RAG2-knockout swine with tumours (**D**). HE-stained lymph nodes of RAG2-knockout swine with metastatic tumour (**E**) and IHC analysis using an anti-GFP antibody (**F**). HE-stained lymph nodes of RAG2-knockout swine (**G**) and IHC analysis using an anti-GFP antibody (**H**). Immunohistochemical analysis of alpha SMA expression in lymph nodes of RAG2-knockout swine with (**I**) and without (**J**) a metastatic tumour. Immunohistochemical analysis of TGF-beta expression in lymph nodes of RAG2-knockout swine with (**K**) or without (**L**) a metastatic tumour. Immunohistochemical analysis of tenascin C expression in lymph nodes of RAG2-knockout swine with (**M**) and without (**N**) a metastatic tumour. The scale bar = 1 mm (**A–D**) and 250 µm (**E–N**).

Lymph nodes of wild-type and RAG2-knockout swine with or without A431-induced tumours were collected and subjected to histological analysis. Lymphoid follicles were observed in wild-type swine (Fig. 5A), although a few aggregated lymphocytes and immature lymphoid follicles were observed in knockout swine (Fig. 5B), as described in our previous study^27^. In swine with metastases, tumour cells replaced the pre-existing lymph node architectures (Fig. 5C). Immunohistochemistry (IHC) using an anti-GFP antibody confirmed the existence of the tumour and the boundary between tumour and lymph nodes (Fig. 5D). High magnification of tumour metastasis detected using hematoxylin and eosin (HE) staining and IHC analysis using an anti-GFP antibody are shown in Fig. 5E,F, respectively. The corresponding analyses of lymph nodes are shown in Fig. 5G,H. The tumour cells showed high-grade nuclear atypia and formed irregular tumour nests (Fig. 5E).

To characterise the microenvironment of this tumour metastasis model, we analysed RAG2-knockout swine with and without metastatic tumours. For this purpose, we performed IHC using antibodies against smooth muscle actin (SMA), transforming factor (TGF)-beta and tenascin C, which serve as markers for cancer-associated fibroblasts, cytokines and the extracellular matrix, respectively. Alpha SMA was expressed at higher levels in involved lymph nodes (Fig. 5I) vs the control (Fig. 5J). TGF-beta was only expressed in cancer cells (Fig. 5K) but not in control lymph nodes (Fig. 5L). Tenascin C was expressed at higher levels (Fig. 5M) compared with control lymph nodes (Fig. 5N).

## Discussion

Here we describe the establishment of a model of metastasis to SLNs using immunodeficient swine. To our knowledge, there are no other reports of using swine or other large animals for this specific purpose. Several related studies of SLNs in swine have been published. For example, ultrasound employing microbubbles can be used to identify SLNs^29^, and SLNs can be identified using Sonazoid^30^. However, these studies employed wild-type swine. Therefore, the results may not be generalized to metastasis to SLNs.

We previously established a rodent model of SLN metastasis that offers the possibility of preoperative rapid diagnosis of SLNs to enable the use of minimally invasive treatments such as photodynamic therapy^23^. Here it was possible to apply clinical identification and extraction methods to study SLNs, which will make possible the experiments aimed to study metastasis in a large animal. An example is provided below.

First, large animal models can confirm the clinical efficacy and safety of new drugs, as well as for the development of innovative therapeutic strategies of lymph node metastasis. Thus, the metastasis model is more suitable for preclinical experiments, because it is possible to target progressive cancers, particularly those with lymph node metastasis. Specifically, photodynamic therapy (PDT) has attracted attention as a minimally invasive treatment. A study of a rodent model of lymph node metastasis employed PDT^25^. In this study, A431 cells were injected to the forelimbs of BALB/c nude mice to develop lymph node metastasis, 2-methacryloyloxyethyl phosphorylcholine -verteporfin was subsequently injected at the dorsum manus, and 75 J of light was delivered to the skin. This combination significantly reduces SLN metastasis compared with the control. The depth of penetration of a laser beam is wavelength-dependent. Therefore, using large animals vs small rodents to test PDT is obviously more applicable to clinical practice. Further, a large animal model provides the ability to study metastasis residing in tumours of visceral organs. Second, large animal models of metastasis can be applied to confirm new SLN verification methods for noninvasive diagnosis. For example, a published method identified SLNs in patients with breast cancer treated with agents such as SPIO nanoparticles^11^. Moreover, imaging methods that assess the pharmacokinetics of SLNs are applied in clinical practice^12,13^. Our swine model likely will prove useful to verify these methods. Further, studies are available that determined the sizes of tin and phytate-isotope colloids particles that specifically accumulate in target lymph nodes in the presence of a consistent lymph flow^31^. Magnetic nanoparticles, 20-nm in diameter, enhance detection of SLNs in combination with magnetic resonance imaging in intact rodent and models of SLN metastasis^23^. For these new SLN identification methods, we consider that our model can verify the optimal size and structural features of the particles. Third, we detected the expression of SMA, TGF-beta and tenascin C, indicating the applicability of our model for studying tumour-stroma interactions.

There are several limitations in the present study. We used A431 cells as we did in previous studies^23,25^. To better assess the clinical relevance of our model, we plan to use cell lines or primary tumour cells derived from other cancers (i.e. breast cancer). Tumour cells were subcutaneously injected into the limbs or auricles to facilitate their entry into the lymphatic flow. Considering the mechanism of lymph flow-mediated lymph node metastasis in clinical practice, this method is feasible and more clinically relevant. However, metastasis to SLNs did not always occur even when tumour growth was confirmed at the implantation site. The size of the primary tumour differed depending on the site, although there was no apparent relationship between tumour size and lymph node metastasis. Therefore, it may be difficult to use this model to analyse processes such as the immune response and metastasis. To effectively apply this model to experiments, it will be necessary to establish a diagnostic method to confirm the presence or absence of metastasis to the SLNs before their removal.

In the present study, we used CT to measure and compare the diameters of lymph nodes. Previous studies employed ultrasound to identify SLNs^29,30^. We believe that combining these diagnostic imaging techniques will more accurately evaluate the presence or absence of metastases. In clinical practice, metastasis is confirmed at the time of SLNB, but it is difficult to diagnose metastases, such as micrometastases, in preoperative images. We plan to establish a micrometastasis model but must consider other methods to detect metastatic tumour cells before the SLNB is performed.

## Methods

### Cell lines

The human epithelial carcinoma cell line A431 (American Tissue Culture Collection, Rockville, MD) was used to generate cells stably transfected with green fluorescent protein (GFP). A431 cells were maintained in Dulbecco’s modified Eagle’s medium with 10% heat-inactivated foetal bovine serum (Gibco, Grand Island, NY) in a humidified 5% (v/v) CO_2_ incubator at 37 °C.

### Swine

We used nine crossbred swine (Landrace, Yorkshire, and Duroc), including two wild-type and seven RAG2 knockouts. The information about the swine are shown in Supplementary Tables 1 and 2. Tumour cells were implanted into six RAG2 knockout swine 12 ± 2-days-old, weighing 2.7 ± 0.4 kg. A431 cells (1 × 10^6^ in 1 mL phosphate-buffered saline) stably expressing GFP were injected into the posterior side of the auricle, forelimb, and hindlimb. Tumour size and swine weight were measured every 2 weeks. Animals were maintained on a standard laboratory chow diet and had free access to tap water. SLNs were extracted at 10 weeks of age (8 weeks after tumour transplantation) when the swine weighed 18.0 ± 2.0 kg. SLNs were observed using a stereoscopic fluorescence microscope and were analysed using HE staining and IHC. National Agriculture and Food Research Organization maintains several heterozygous RAG2-knockout males and females. Therefore, we can regularly produce homozygous RAG2-knockout swine by simple matings to insure the reproducibility of our experiments. We used swine of approximately the same age and size.

The Institutional Animal Care and Use Committees of Keio University (Approval number: 8073) and the University of Tokyo (Approval number: 986-2732) approved this study. All animal experiments were performed in accordance with the local ethics law, the regulations of the local ethics committee and Institutional Guidelines on Animal Experimentation at Keio University.

### CT

CT was performed after the swine were administered general anaesthesia. The whole body was scanned without a contrast medium (1-mm slices).

### Surgical and histological procedures

Indigo carmine dye or ICG was used to detect SLNs in the same manner applied in clinical practice^9,10^. Indigo carmine (1 mL) or ICG (0.5 ml) was injected into the posterior sides of the auricle, forelimb and hindlimb, and SLNs were excised 15 min later. Incisions were made in the groin and neck to follow the lymph flow. Blue lymph nodes were defined as SLNs and removed. During the procedures, sedation was induced by intramuscular injection of medetomidine and butorphanol (1 mL per animal, each), and anaesthesia was maintained using 1.5–2% isoflurane after intubation. SLNs were observed using a stereoscopic fluorescence microscope and pathologically evaluated.

### Fluorescence microscopy

A Nikon stereoscopic fluorescence microscope SMZ25 (objective lens, SHR Plan Apo 0.5×) was equipped with a Nikon DS-Ri2 camera. Images were processed using NIS-Elements D (Nikon Corporation, Minato, Japan).

### IHC

Specimens were deparaffinized in xylol, rehydrated in a descending series of ethanol concentrations and antigens were retrieved using Target Retrieval Solution (Dako Japan, Tokyo Japan) at 121 °C for 10 min. After inactivated with endogenous peroxidase in 0.5% periodic acid for 10 min, specimens were blocked in 4% skimmed milk in TBS for 30 min. Antibodies (all from Dako Japan, Tokyo) against GFP (1:100, ab183734), alpha SMA (1:1000, ab7817), TGF-beta (1:100, ab190503) and tenascin C (1:100, ab108930) were incubated overnight at 4 °C, washed with TBS for 30 min and then incubated with ENVISION (Dako Japan, Tokyo, Japan) for 30 min. After washing with TBS for 30 min, immune complexes were visualised using DAB for 1–5 min, counterstained with hematoxylin for 1 min and then dehydrated and mounted on coverslips. The images were acquired using a NanoZoomer (Hamamatsu photonics K.K., Shizuoka, Japan).

## Supplementary information

Supplementary Information

## Data Availability

All the data used in this paper are available.
