# Supplementary material for: Establishment of a model of sentinel lymph node metastasis using immunodeficient swine
Source: Sci Rep. 2019 May 28;9:7923. doi: 10.1038/s41598-019-44171-w (PMC6538626; doi:10.1038/s41598-019-44171-w)
Supplement: Supplementary file 1 — Supplementary Information [file 41598_2019_44171_MOESM1_ESM.docx]

**Supplementary Information**

**Establishment of a model of sentinel lymph node metastasis using immunodeficient swine**

Toshiaki Kurihara^1^, Sachiko Matsuda^1^*, Yuki Nakamura^1^, Shunichi Suzuki^2^, Daiichiro Fuchimoto^2^, Akira Onishi^3^, Kohei Saeki^4^, Takayuki Nakagawa^4^, Reina Fujiwara^5^, Masatoshi Kamata^5^, Junko Kuramoto^6^, Kaori Kameyama^7^, Masaki Sekino^8^, Moriaki Kusakabe^9, 10^, Tetsu Hayashida^1^, Hiromitsu Jinno^11^ and Yuko Kitagawa^1^

^1^Department of Surgery, Keio University School of Medicine, 35 Shinanomachi, Shinjuku, Tokyo 160-8582, Japan

^2^Division of Animal Sciences, Institute of Agrobiological Sciences, National Agriculture and Food Research Organization (NARO), Tsukuba, Ibaraki 305-0901, Japan

^3^Laboratory of Animal Reproduction, Department of Animal Science and Resources, College of Bioresource Sciences, Nihon University, Fujisawa, Kanagawa 252-0880, Japan

^4^Graduate School of Agricultural and Life Sciences, The University of Tokyo, 1-1-1 Yayoi, Bunkyo, Tokyo 113-8657, Japan

^5^Veterinary Medical Center, Graduate School of Agricultural and Life Sciences, The University of Tokyo, 1-1-1, Yayoi, Bunkyo, Tokyo 113-8657, Japan

^6^Department of Pathology, Keio University School of Medicine, 35 Shinanomachi, Shinjuku, Tokyo 160-8582, Japan

^7^Department of Diagnostic Pathology, Keio University Hospital, 35 Shinanomachi, Shinjuku, Tokyo 160-8582, Japan

^8^Graduate School of Engineering, The University of Tokyo, 7-3-1 Hongo, Bunkyo, Tokyo 113-8656, Japan

^9^Graduate School of Agricultural and Life Sciences, Research Center for Food Safety, The University of Tokyo, 1-1-1 Yayoi, Bunkyo, Tokyo 113-8657, Japan

^10^Matrix Cell Research Institute, Inc., 1-35-3 Kamikashiwada, 300-1232 Ushiku, Ibaraki, Japan

^11^Department of Surgery, Teikyo University School of Medicine, 2-11-1 Kaga Itabashi, Tokyo 173-8605, Japan

*Address correspondence to this author at the Department of Surgery, School of Medicine, Keio University, 35 Shinanomachi, Shinjuku, Tokyo, Japan. Tel: +81-3-3353-1211; Fax: +81-3-3355-4707; E-mail: [matsuda-sa@umin.ac.jp](mailto:matsuda-sa@umin.ac.jp)

Table S1. Data of work done on all swine

|  | 1 | 2 | 3 | 4 | 5 | 6 | 7 | 8 | 9 |
| --- | --- | --- | --- | --- | --- | --- | --- | --- | --- |
| Genotype | wild type | wild type | RAG2-KO | RAG2-KO | RAG2-KO | RAG2-KO | RAG2-KO | RAG2-KO | RAG2-KO |
| Tumour injection | - | - | - | + | + | + | + | + | + |
| CT scan | - | + | + | - | + | + | + | + | - |
| Photograph | + | - | + | - | - | - | - | - | + |
| Lymph node dissection | + | + | + | + | + | + | + | + | + |
| Measurement of body weight | - | - | - | - | - | + | + | + | - |
| Measurement of tumour diameter |  |  |  | + | - | + | + | + | - |

Table S2. With or without tumour engraftment and lymph node metastasis in RAG2-KO swine

|  |  | 4 | 5 | 6 | 7 | 8 | 9 |
| --- | --- | --- | --- | --- | --- | --- | --- |
| Tumour engraftment | right auricle | + | + | - | - | - | + |
|  | left auricle | + | + | - | - | + | + |
|  | right forelimb | ND | ND | - | - | + | + |
|  | left forelimb | ND | ND | + | + | + | + |
|  | right hindlimb | + | + | - | - | - | + |
|  | left hindlimb | + | + | - | - | - | + |
| Lymph node metastasis | right parotid lymph nodes | + | - | - | - | - | + |
|  | left parotid lymph nodes | + | - | - | - | + | + |
|  | right superficial cervical lymph nodes | ND | ND | - | - | - | + |
|  | left superficial cervical lymph nodes | ND | ND | - | - | - | + |
|  | right superficial inguinal lymph nodes | - | + | - | - | - | + |
|  | left superficial inguinal lymph nodes | + | - | - | - | - | + |

ND: not determined
